# ChatGPT in finance: Applications, challenges, and solutions

**DOI:** 10.1016/j.heliyon.2024.e24890

**Published:** 2024-01-17

**Authors:** Muhammad Salar Khan, Hamza Umer

**Affiliations:** a Schar School of Policy and Government, George Mason University, USA; b Hitotsubashi Institute for Advanced Study (HIAS), Institute of Economic Research (IER), Hitotsubashi University, Japan

**Keywords:** ChatGPT, Finance, Ethical challenges, Policies, Applications, Artificial intelligence

## Abstract

The emergence of ChatGPT, a generative artificial intelligence tool, has sparked a revolution in the finance industry, enabling individuals to interact with technology in natural language. However, the use of ChatGPT in finance presents a profound array of ethical considerations that demand careful scrutiny to ensure its responsible and ethical use. After a concise exploration of ChatGPT's applications in finance, this policy article delves into the ethical challenges arising from the use of ChatGPT in finance, including outcomes contaminated with biases, incorporation of fake information in the financial decisions, concerns surrounding privacy and security, lack of transparency and accountability in the decision-making processes and financial services, human job displacement, and the intricate web of legal complexities. Our article asserts that financial institutions employing ChatGPT must proactively devise strategies to confront these burgeoning challenges, mitigating their adverse effects on both individuals and society as a whole. Additionally, we propose relevant policies to tackle these ethical quandaries head-on. In essence, this article illuminates the imperative need for a meticulous ethical framework, facilitating an informed and responsible use of ChatGPT in the realm of finance, safeguarding the welfare of individuals and society. While our work significantly contributes to the research and practice of finance, we also identify future research avenues.

## 1 Introduction

The development of large language models (LLMs) has been a noteworthy achievement in generative artificial intelligence (AI), exemplified by ChatGPT, a prominent innovation by OpenAI, using the Generative Pre-training Transformer (GPT) architecture [1]. Essentially a deep learning algorithm, ChatGPT boasts a remarkable ability to autonomously learn from data, generating sophisticated text outcomes post training [2]. The GPT series, from the initial GPT-1 trained on 117 million parameters to the latest GPT-4 with 1.76 trillion parameters [3], demonstrates the evolution of ChatGPT technology, enabling it to generate human-like responses [[4], [5], [6]] and even work with image inputs [7]. Outperforming most humans on a battery of academic and professional exams, ChatGPT's potential applications are diverse, ranging from natural language processing to the development of chatbots and virtual assistants [8]. It excels in its capacity to learn and generate text in multiple languages and across a wide array of topics. As a powerful AI tool, ChatGPT holds the potential to revolutionize the operations of businesses and industries, particularly within the finance sector. Unlike existing financial innovations such as digital accounting systems [9] and FinTech [10], which focus on automating financial services and records, ChatGPT's distinguishing feature lies in its expertise in natural language understanding and generation, rendering it suitable for text-based interactions and tasks.

However, as with any new technology, ChatGPT is not flawless; its evolution and inherent limitations can give rise to ethical considerations and challenges that must be addressed to ensure its responsible use for the wellbeing of both individuals and society [[11], [12], [13]]. While the literature on challenges of ChatGPT in domains such as health and medicine, education, and scientific research [11,14,15] has expanded rapidly, to the best of our knowledge, the literature focusing specifically on the challenges of ChatGPT in finance is limited. Most studies in this domain have primarily explored issues of using ChatGPT for academic research in finance [5,16] leaving the challenges related to real financial decisions largely unexplored. While academic research in finance is important, accurate and well-informed financial decisions are equally crucial due to their profound impact on the economic wellbeing of individuals and society.

Moreover, as ChatGPT infiltrates the realm of actual financial decision-making, it becomes imperative to identify and study the potential challenges that could result in inaccurate financial decisions and cause a loss of scarce time and money. Identification and a thorough discussion of these challenges can serve to alert the general public about negativities of using ChatGPT in real financial decisions and offer valuable feedback to the relevant authorities, enabling them to work towards mitigating these challenges for a safe and fruitful use of technology in financial decision-making. To bridge the aforementioned gap in the literature, this study examines the ethical challenges of using ChatGPT in finance. To do so, the study performs a deductive analysis based on the existing literature and using our own critical evaluation of ChatGPT's role in finance, identifying six important challenges.

First, it is highly probable that ChatGPT can incorporate gender, racial, political, and recency biases in the financial output, leading to inaccurate and undesirable financial outcomes. Second, there is a risk that ChatGPT can incorporate sophisticated and hallucinatory fake information that may be difficult to detect in the suggested financial output. The presence of such information can lead to acute financial losses. Third, there is a risk that financial and personal information of both individuals and organizations used by ChatGPT could be hacked and accessed by detrimental parties, putting both financial and personal information at stake. Fourth, there are uncertainties surrounding transparency and accountability in the tool's decision-making processes, which can reduce confidence in the tool, and at the same time, make it difficult to navigate through potential legal problems linked to the financial outcomes. Lastly, the automation of financial tasks (such as accounting and marketing) can lead to human replacement and job loss. The net benefits to society might turn out to be negative in the case of large-scale human replacement in the financial industry. Therefore, we need to plan ahead of time to adjust individuals being replaced by ChatGPT in other relevant financial roles.

Our work makes several contributions to literature in the field of generative artificial intelligence (AI) and finance. First and foremost, we address a significant gap in existing research. While research studies focusing on ChatGPT have expanded rapidly [8,11,13], the literature focusing on the ethical considerations surrounding ChatGPT's integration into the financial domain is relatively scarce [5]. This scarcity of insight is a matter of concern for various stakeholders, including financial institutions, regulators, policymakers, and researchers. By examining the ethical challenges specific to ChatGPT's use in finance, our paper not only sheds light on issues that have been underrepresented but also prompts a crucial dialogue on responsible use of ChatGPT (or generative AI deployment) within the financial sector.

Furthermore, our work extends beyond academic discourse to have practical implications. We aim to raise awareness among financial institutions, regulators, and policymakers about the potential risks associated with ChatGPT in finance. While the financial industry increasingly embraces AI technologies, the ethical dimensions of this integration cannot be ignored. By identifying and discussing these ethical challenges, we provide a foundation for informed decision-making and emphasize the pressing need for the development of robust policies and regulations governing ChatGPT's use. Our contributions, therefore, go beyond mere academic inquiry; they underscore the urgency of ensuring ethical generative AI practices within the financial sector and pave the way for more in-depth research into these challenges and their practical implications.

The rest of the article is structured into eight sections. Section 2 briefly discusses the possible uses of ChatGPT in finance. Section 3 delves into the ethical challenges that may arise from ChatGPT's use in finance. Section 4 offers relevant solutions to tackle these issues. Section 5 mentions limitations of the analysis, whereas section 6 highlights the research and practical implications of the study. The last two sections make recommendations for future research and conclude the paper, respectively.

## 2 Methodology

As our work closely aligns with a conceptual policy paper, our methodology relies on a review of literature to deduce possible ethical challenges associated with ChatGPT. We do not conduct a quantitative investigation due to a lack of available data on ChatGPT. While most available preliminary studies report outcomes based on prompts given to ChatGPT [13,14], we refrain from such an analysis because findings derived from a limited number of prompts lack generalizability. Even if we elicit multiple responses from ChatGPT for the same prompt, a lack of general consensus on evaluating these responses, especially in the case of assessing biased outcomes, can lead to contradicting results [17].

Additionally, since ChatGPT uses data fed into the World Wide Web (www) and the tool is influenced by a recency bias, its response to the same prompt can vary. Thus, concerns regarding replication arise with a prompt-based methodology. Therefore, to avoid these pitfalls, we provide a deductive analysis.

To deduce our main findings, we primarily rely on existing prominent studies on ChatGPT. As ChatGPT is a new tool and is still evolving, only a segment of the available research has undergone the scientific peer review process, and several studies are available as working papers. To draw insights from a broader set of studies, we mainly depend on published studies but use working papers wherever we find them relevant and necessary. Furthermore, in most cases, we support our arguments with relevant examples from literature. However, in certain cases, to the best of our knowledge, the potential challenges of ChatGPT in finance we point out have not been tested with the help of data so far. Therefore, it is difficult to identify and cite the relevant examples for such instances. Despite this limitation, identifying potential undetected challenges is imperative to ensure the general public understands them, and relevant authorities could address them in the future.

## 3 Functions of ChatGPT in finance

Before delving into the ethical challenges posed by ChatGPT in finance, it is useful to provide a brief overview of ChatGPT's utility in the financial domain. ChatGPT boasts vast language processing capabilities, enabling it to perform a range of functions. Specifically, within the business and finance sphere, ChatGPT finds applications in a multitude of roles. Some of these applications include understanding the dynamics of the financial market [18], offering customer service and suggesting products [19], performing named entity recognition [20], generating financial summaries, reports, and forecasting financial outcomes [21], as well as providing personalized investment recommendations [12]. ChatGPT can also potentially be trained for fraud detection and prevention. More details about these functions of ChatGPT are summarized in Table 1 below.

**Table 1:** Functions of ChatGPT.

Function	Description	Example
A) Understanding the dynamics of the financial market	ChatGPT is used to classify financial news and perform text-based and social media-based analyses to gain insights into the financial market	Identifying trends and predicting future market movements based on news articles and social media posts
B) Customer service	ChatGPT can be trained to interact with customers and answer their questions about various financial products and services	Providing information on banking hours, loan terms, and suggesting financial products
C) Named entity recognition	ChatGPT can extract and understand large amounts of financial information from unstructured texts	Identifying names of companies, people, and financial products mentioned in a news article
D) Generating financial summaries, reports, and forecasting outcomes	ChatGPT can generate financial summaries, reports, and predict financial outcomes	Generating a financial report that summarizes a company's performance in the previous quarter
E) Personalized investment recommendations	ChatGPT can make personalized investment recommendations based on the financial objective and risk-preferences of individuals	Recommending a diversified portfolio for a customer with a low-risk tolerance
F) Fraud detection and prevention	ChatGPT can be trained for fraud detection and prevention	Analyzing customer data and financial transactions to detect fraudulent activity

## 4 Ethical challenges of using ChatGPT in finance

While ChatGPT offers numerous benefits, including improved efficiency, accuracy, and personalization of financial services, it also raises important questions about privacy, security, and fairness. In this section, we will briefly outline some of the most pressing ethical challenges associated with ChatGPT in finance.

### 4.1 Biased outcomes

ChatGPT relies on large data sourced from the internet to provide output. When existing internet data about a financial aspect contain biases, such as those related to gender, religion, race, politics, region or other such factors, ChatGPT may inadvertently incorporate and amplify these biases in its responses [22]. This unintended consequence can lead to undesirable outcomes and potentially perpetuate the existing biases on a larger scale, given the tool's widespread availability and accessibility.

Preliminary evidence shows the presence of bias in ChatGPT's responses. For instance, when tasked with creating a poetry (limerick), ChatGPT favored liberal politicians over conservatives in Ireland [23]. Similarly, in a question repeated 100 times, ChatGPT displayed a preference for left-leaning political stances in Brazil, the United States, and the United Kingdom [17]. Additionally, based on an analysis of 630 political questions, ChatGPT exhibited a bias towards left-wing and pro-environmental politics in Germany and the Netherlands [24]. These initial findings suggest that ChatGPT may also show similar biases in its responses related to financial organizations aligning with specific political spectrums or engaged in particular businesses that are environment friendly.

Moreover, since ChatGPT draws information from media sources, it may more frequently and easily identify and suggest firms with robust media coverage through advertising, even though those firms financially might not be an ideal match for the given prompt. Furthermore, as smaller firms do not have same amount of information and data available online compared to the larger firms, using ChatGPT to conduct due diligence can lead to incorrect evaluation of the smaller firms, and subsequently, might suggest inefficient investment opportunities to potential investors [25]. All this underscores the need for vigilant scrutiny and measures to mitigate biases in ChatGPT's financial output.

### 4.2 Fake information and misinformation

Fake financial news, misinformation, and sham businesses have become pervasive issues with far-reaching global consequences. While ChatGPT has been designed to identify and filter out fake information [26], there are still concerns about its ability to ensure the credibility of the information it processes. Despite undergoing extensive data training, ChatGPT may unintentionally incorporate fake news into market sentiment analysis or financial projections, resulting in inaccuracies. Furthermore, the ever-evolving nature of fake information necessitates continuous training for ChatGPT to identify and eliminate emerging sources of misinformation.

ChatGPT's ability to generate consumer recommendations also carries inherent risks, as it may sometimes provide false, fake, or hallucinatory information. As a result, the use of ChatGPT for purposes such as collecting product information, arranging marketing campaigns, and constructing personalized investment choices can be misleading [22,27]. This misinformation can heighten financial risks for both consumers and investors, potentially eroding trust in the tool's application in the realm of finance. Moreover, ChatGPT's recommendations may inadvertently exhibit a bias towards pro-environment products and organizations, mirroring its inclination towards pro-environmental groups [24], which could further result in a loss of trust from other interest groups.

While ChatGPT can be used to extract and summarize financial information and construct financial statements, this practice can be problematic because ChatGPT can make factual mistakes when dealing with numerical data. For instance, as demonstrated by Van Dis and colleague researchers, generating a summary of their own published systematic review using ChatGPT resulted in inaccuracies [2]. Specifically, ChatGPT-generated summary incorrectly states that the review article is based on 46 studies, while it actually uses 69 studies. Moreover, the summary also overstates the findings reported in the review article. This highlights the possibility of ChatGPT making factual errors when extracting information from online documents, and this is more likely to happen in the case of financial documents because they are generally rich in numbers. Therefore, both summarizing and generating financial documents carry inherent risks and could lead to financial losses if decisions are based on factually incorrect outputs generated by ChatGPT.

### 4.3 Privacy concerns

ChatGPT relies on an extensive repository of financial data, including both individual and organizational transactional information. Therefore, the privacy of both individuals and organizations can be compromised if these data are accessed by malicious third parties [22]. Furthermore, the mishandling of private financial information can cause financial harm to individuals. Malicious actors may jailbreak ChatGPT to generate phishing attacks [28], impersonate firms or individuals, and produce convincing but fraudulent emails to conduct financial scams. All this also renders ChatGPT an attractive target for cybercriminals. They can swiftly generate codes at a faster pace and modify them to attack the security walls of financial institutions and individuals. Furthermore, they can obfuscate such codes to evade detection by security tools. Additionally, the widespread availability of ChatGPT makes it a valuable resource for novice cybercriminals, enabling them to easily generate codes that can be modified with minimal human effort and could be used to cause financial damage. These activities not only pose serious financial threats but also engender psychological unrest for individuals, and can damage the reputation of firms [29].

Although ChatGPT follows certain protocols to ensure privacy and security [26], it is imperative that these protocols are transparently shared with both financial institutions and individuals engaged in financial transactions. This transparency creates a well-understood and clear environment where all stakeholders are aware of the measures in place to safeguard their privacy and data. Moreover, in the event of a data breach, it is crucial that robust preventive and mitigation measures are firmly established and widely disseminated. This dissemination is pivotal in maintaining societal trust in the use of AI-driven tools, such as ChatGPT in finance. Demonstrating a proactive approach to handling privacy and data breaches and promptly addressing any such incidents is integral to upholding trust and confidence in the responsible use of these advanced technologies.

### 4.4 Transparency and accountability

Another major ethical consideration when using ChatGPT in finance revolves around the imperative need for transparency and accountability, particularly in light of regulations such as the European General Data Protection Regulation (GDPR) and the White House's Office of Science and Technology Policy (OSTP) Blueprint for AI Bill of Rights [30,31]. ChatGPT relies on complex AI algorithms, specifically Natural Language Processing (NLP) [32], to make decisions, but these algorithms can often be opaque [33]. This opacity often makes it challenging to comprehend the process through which the technology reaches its conclusions [27,34].

Similar to all AI systems, ChatGPT functions with imperfect and noisy data to generate outcomes. However, as with any AI tool, ChatGPT sometimes fails to deliver by producing an incorrect output [2]. ChatGPT's algorithms rely on a “training phase,” wherein it learns from human-labeled datasets, using these examples to enhance its performance before handling non-labeled data. Therefore, even after training, ChatGPT may mislabel or miscategorize new instances of data sourced from the internet that it has never encountered before. While such errors are acceptable in the tech realm as the tool matures, in the financial world, erroneous decisions or faulty suggestions by ChatGPT could have severe repercussions for customers and organizations. In the event of adverse outcomes, ChatGPT owes an explanation to customers and organizations alike. Demands for accountability from ChatGPT will encourage the tool to become more transparent and responsible in terms of its design and decision-making.

### 4.5 Human replacement

ChatGPT has the potential to automate dozens of routine tasks within the finance sector, such as accounting, tax filing, record-keeping, marketing, data analysis and forecasting, among others. This massive versatility could unfortunately lead to the replacement of millions of human workers in the industry [27,35,36].

This development gives rise to a significant ethical concern, akin to the dilemmas posed by emerging technologies like robotics – the issue of human replacement. While technology can efficiently automate many tasks, it falls short in replicating the nuanced facets of human judgment and intuitive decision-making. These human attributes are indispensable for ethical decision-making in many aspects of the finance sector. Therefore, it is necessary to carefully consider the implications of ChatGPT's automation of tasks and the impact it could have on the workforce. This consideration extends beyond the realms of efficiency and productivity, delving into the preservation of ethical values and human involvement in critical decision-making processes within finance.

### 4.6 Legal issues

The training and learning process of ChatGPT occurs on a global scale, and therefore, the financial outcomes, including investment decisions affecting both individuals and organizations, may potentially infringe domestic regulations and laws. Furthermore, the content generated for business purposes, such as marketing materials and financial reports, can exhibit substantial similarities across different firms within the same industry [27]. In the event of publication, these materials could lead to issues of piracy and copyright infringement.

Complicating matters further, as these outputs are generated by a machine, the resolution of such cases becomes considerably more challenging within the legal system. This is primarily due to a lack of relevant rules and laws, owing to the nascent nature of ChatGPT and similar AI technologies. Furthermore, as financial laws are heterogenous across countries, the cross-country legal problems originating due to ChatGPT's financial output could be difficult to resolve due to their heterogenous laws. Consequently, this scenario engenders acute legal challenges not only for financial organizations, but also for legal systems tasked with addressing the evolving complexities presented by AI-driven outputs in the finance industry.

## 5 Possible solutions to the challenges

There can be multiple paths to achieve the same end goals of mitigating the ethical challenges of ChatGPT in finance. We here discuss five broad mechanisms that can be helpful in mitigating the ethical challenges discussed above.

a) The issue of biased outcomes can be mitigated by ensuring that the data used for further training of ChatGPT is devoid of these biases. Moreover, ChatGPT developers (OpenAI) and representatives from the general public should work together to develop future algorithms that mitigate biases in the outcomes generated by ChatGPT. Government regulators and consumer protection bureaus can oversee to streamline these interactions, and to ensure they lead to productive outcomes. Such a participatory approach, explained in-depth in a recently published study on ChatGPT [37] and previously elaborated in the context of ethical use of emerging biotechnology [38], adheres to an inclusive process, enabling ChatGPT to implement an algorithm that embraces diverse financial perspectives and effectively mitigates biases in its outcomes.
b) To tackle the challenge of fake and misinformation, we think of multiple solutions. First, during the algorithm development stage, the developers can specify a robust framework or algorithm for ChatGPT to extract information from only credible and pure sources. Second, human supervision in the form of randomly auditing the generated outcomes can be an effective tool to identify whether fake information or misinformation is being used by ChatGPT. Subsequently, algorithms can be modified to ensure such information sources are not used again in future financial recommendations and other financial analyses. Furthermore, the involvement of human supervision will create new job opportunities, and to some extent, mitigate job losses due to ChatGPT-driven automation in the financial sector.
c) To ensure that the sensitive financial data of organizations and individuals are not compromised by malicious actors targeting ChatGPT, we need to practice caution at the organizational level by taking the following possible precautions. First, organizations should establish a clear policy about the nature and extent of their own as well as their client's individual financial data that can be safely accessed by ChatGPT. As the nature of produced data varies across industries, each industry is best positioned to make such policies independently and ensure their complete implementation. These policies should be clearly and effectively disseminated among the general public to ensure they understand what sort of financial transactional data might be available to ChatGPT. Second, public regulators can collaborate with industries to ensure the formulation and uniform implementation of such policies within a certain time. Moreover, the push from the regulators can also help in achieving these goals at a faster pace, and therefore, minimize the possibility of financial loss for individuals and organizations. Lastly, organizations should constantly update their data security to safeguard themselves from cybercriminals and hackers. Using multiple security walls and frequently changing them would make it difficult for hackers to breach, even if they obtain help from ChatGPT to construct malicious codes.
d) The legal issues due to use of ChatGPT in finance require a globally coordinated effort for their solution. The countries such as the United States, the United Kingdom, Japan, China and the European Union and international bodies such as UNESCO and the World Bank's ICSID should collaborate to design a comprehensive legal framework to tackle both domestic and cross-country legal issues that can arise by using ChatGPT in finance. Establishing a dedicated body, such as ‘Global AI Regulator and Court of Arbitration,’ can be an effective way forward to deal with legal issues of using ChatGPT in finance. This body would also develop global standards to ensure that ChatGPT's applications in finance adhere to universally accepted norms. These standards can lead to a consensus on the permissible and ethical use of ChatGPT in finance, facilitating the resolution of cross-country financial disputes, such as issues related to piracy and plagiarism in financial documents and similar marketing campaigns produced by ChatGPT. Moreover, the establishment of a global body is necessary to ensure the adoption of minimum privacy and security protocols, measures to mitigate biased outcomes, and systems to combat fake and false information by all nations employing ChatGPT and other such AI technologies. These efforts will promote a unified and uniform advancement in the application of ChatGPT in finance, setting a precedent for the development of similar protocols in other non-financial domains.
e) To ensure accurate and reliable financial decisions, it is crucial to acknowledge and leverage both the strengths of AI-based decision-making by ChatGPT and the intuitive decision-making of finance professionals based on their experiential knowledge. Exclusive reliance on ChatGPT algorithms can result in the loss of valuable insights from finance professionals. Therefore, a hybrid approach combining both types of decision-making is recommended. Professionals can provide their own perspectives while ChatGPT algorithms analyze the data. This approach enables a comparison of decisions made by both ChatGPT and humans, fostering mutual learning and improvement. Moreover, human involvement can provide oversight over the financial decisions suggested by ChatGPT, and subsequently, lower the incidence of incorporating flawed suggestions made by ChatGPT. It can also address legal barriers in the financial domain that ChatGPT might overlook while proposing financial solutions to the users. Furthermore, human involvement can, to some extent, delay or mitigate the immediate threat of job displacement caused by ChatGPT. Finally, human involvement in overseeing and assessing ChatGPT's performance in finance may also create new job opportunities in this evolving landscape.

As ChatGPT evolves over time, the existing ethical issues in the domain of finance will also evolve, and new problems will emerge. To counter these challenges, the above suggested solutions will have to be updated and modified constantly for sustained financial wellbeing of individuals, organizations, and society as a whole.

## 6 Limitations of our analysis

It is important to discuss the limitations of the existing study. First, our analysis, focusing on the challenges of using ChatGPT in finance, is mostly based on the outcomes of the existing studies, which, in turn, use a limited number of observations to deduce conclusions. A comprehensive analysis based on large data could provide richer insights into these challenges. However, the lack of large-scale data for the time being makes it difficult to conduct such a systematic analysis. Second, our proposed solutions are empirically untested, and therefore, the effectiveness of these solutions remains unclear. The relevant actors implementing these solutions should apply them carefully considering their contextual factors.

## 7 Research and practical implications

Despite these limitations, our work significantly contributes to the convergence of generative AI and finance by addressing a notable gap in the existing literature. As mentioned earlier, research studies on ChatGPT have proliferated, but there is a relative scarcity of literature focusing on the ethical considerations of its integration into the financial domain—a concern shared by financial institutions, regulators, policymakers, and researchers alike. Our work delves into the ethical challenges specific to ChatGPT's use in finance, catalyzing a crucial dialogue on the responsible deployment of ChatGPT and generative AI within the financial sector. While our deductive analysis is based on the existing literature and using our own critical evaluation of ChatGPT's role in finance, we also present suggestions for future research in the following section of our work, which we think are crucial.

Moreover, our work extends beyond academic discourse, offering practical implications by raising awareness among financial institutions, regulators, and policymakers about potential risks associated with ChatGPT in finance. As the financial industry increasingly adopts AI technologies, it becomes imperative to acknowledge and address the ethical dimensions of this integration. Through the identification and discussion of these ethical challenges, we lay the groundwork for informed decision-making, emphasizing the urgent need for developing robust policies and regulations governing ChatGPT's use. Our contributions extend beyond academic inquiry, highlighting the urgency of ensuring ethical generative AI practices within the financial sector and providing a foundation for more in-depth research into these challenges and their practical implications.

We should note that all the outlined ethical challenges bear significant repercussions and implications. For instance, when financial companies employ ChatGPT without ensuring transparency in its use and without the capability to provide clear explanations for the decisions it generates, they risk facing difficulties in establishing trust with their clients. In an era where accountability and ethical AI deployment are paramount concerns, both clients and regulators demand transparency in AI-driven decision-making processes within the finance sector. Therefore, it becomes not only advisable but essential to establish well-defined guidelines and protocols that prioritize transparency and accountability in ChatGPT's deployment within financial operations. These measures would serve to instill confidence among clients, regulatory bodies, and stakeholders while reinforcing the responsible and ethical use of AI technologies in finance.

## 8 Future research agenda

Future research in this domain should continue to explore and devise methodologies for identifying and mitigating biases in ChatGPT's financial outcomes, with a specific focus on potential socio-religious and other biases. Alongside these biases and challenges, a myriad of other ethical considerations, such as equity and inequality concerns in the local and global use of this technology, warrant thorough investigation. Another avenue of research could focus on operationalizing and quantifying these biases associated with ChatGPT's use in finance. Applied computer scientists might also contribute by developing small LLMs using only pure data. Moreover, research could delve into the development of more robust security and privacy protocols for ChatGPT to safeguard user data in financial contexts. Further investigations into human-AI collaboration in finance, particularly regarding the optimal balance between automated decision-making and human expertise, would also be beneficial. Furthermore, examining the legal implications and developing frameworks to address regulatory challenges in different financial jurisdictions when deploying AI tools like ChatGPT would constitute a fruitful avenue for future research. Finally, future research should judge the severity of the challenges posed earlier based on large-scale data from ChatGPT's use in finance. Data-driven analysis can provide an accurate picture of the challenges, their severity, and hence can help in making relevant policies.

## 9 Conclusion

While ChatGPT has caused a positive disruption in financial decision making, it has concurrently brought forth a host of ethical challenges. The article delved into these challenges, including biases in outcomes, the potential inclusion of fake information in financial planning, concerns regarding security and privacy, the absence of transparency and accountability in decision-making processes, the looming specter of human replacement through automation, and the legal complexities that may emerge in case ChatGPT-generated outcomes clash with a country's financial laws.

To effectively address these multifaceted challenges, it is important for companies, agencies, and financial institutions employing ChatGPT to actively engage in a dialogue with a wide range of stakeholders. This includes the public, experts, regulators, and tech firms, as exemplified in the participatory approach discussed here and drawing upon previous works. We also suggest implementing a robust algorithmic framework to counter hallucination and misinformation. To combat the misuse of data by cybercriminals and other hackers, we recommend organizational-level efforts regarding data availability in the public domain and the establishment of security protocols, among others. Furthermore, at the global level, clear and pertinent standards must be established to navigate the far-reaching legal implications of financial decisions proposed by ChatGPT.

Finally, we recommend adopting a hybrid approach to decision-making, where financial professionals and ChatGPT can complement each other rather than compete. We believe that implementing these basic but essential policy approaches can enhance the tool's societal benefits and foster greater public trust in using it for financial decisions.

While future work will comprehensively tackle the problems we identified, our work is crucial in the ongoing debate on AI ethics and AI innovation. This is especially relevant in light of recent advancements such as the establishment of AI Safety Institute in the United Kingdom, the recently proposed AI Safety Institute in the United States under the latest White House Executive Order on AI, and the global discourse on AI safety and accountability spearheaded by various international bodies such as the OECD and UNESCO. In this time and period where AI innovation is shaping various industries, not just finance, engagement from the research community is crucial more than ever.

## Funding statement

This research did not receive any specific grant from funding agencies in the public, commercial, or not-for-profit sectors.

## Data availability statement

Data included in article referenced in article.

## Ethics declaration statement

Review and approval by an ethics committee were not required for this study because it is a policy concept paper that did not involve any data collection or interaction with humans, and the findings are supported by secondary data.

## Additional information

No additional information is available for this manuscript.

## CRediT authorship contribution statement

**Muhammad Salar Khan:** Writing – review & editing, Writing – original draft, Project administration, Methodology, Investigation, Formal analysis, Conceptualization. **Hamza Umer:** Writing – review & editing, Writing – original draft, Project administration, Methodology, Investigation, Formal analysis, Conceptualization.

## Declaration of competing interest

The authors declare that they have no known competing financial interests or personal relationships that could have appeared to influence the work reported in this paper.
